# P41 Prevalence and antimicrobial resistance among Gram-negative organisms isolated from a tertiary hospital in Guyana (South America)

**DOI:** 10.1093/jacamr/dlaf118.048

**Published:** 2025-07-14

**Authors:** Taudgirdas Persaud, Shazeema Shaw, Joanna Cole Breems, Michael Olabode Tomori

**Affiliations:** Department of Internal Medicine, Georgetown Public Hospital Corporation, Georgetown, Guyana; Department of Internal Medicine, Georgetown Public Hospital Corporation, Georgetown, Guyana; Department of Medical Education and Clinical Sciences, Washington State University, College of Medicine, WA, USA; Department of Public Health, Texila American University, Georgetown, Guyana

## Abstract

**Background:**

AMR continues to pose a significant global public health threat as deaths due to AMR are estimated to reach 10 million lives by 2050, and South Asia, Latin America, and the Caribbean region are forecasted to have the highest AMR all-age mortality rate by 2050. In 2019, Guyana had the 59th highest age-standardized mortality rate per 100 000 population associated with AMR across 204 countries and the 2nd highest in the Caribbean region. Despite these figures, there has never been any comprehensive analysis of the prevalence and antimicrobial resistance patterns of bacteria done in Guyana.

**Objectives:**

To conduct the first comprehensive report of the prevalence and antimicrobial resistance patterns of Gram-negative bacteria and WHO-priority pathogens in clinical isolates obtained at the Georgetown Public Hospital Corporation (GPHC) in Georgetown, Guyana.

**Methods:**

This is a retrospective, descriptive, cross-sectional study of bacterial culture and susceptibility data from January 1 through December 31 of 2023. Culture isolates were identified using the VITEK 2 system, and resistance profiles were interpreted according to the Clinical and Laboratory Standards Institute (CSLI) guidelines. WHONET software was used to analyse the prevalence and resistance profiles of Gram-negative organisms. Prevalence of third-generation cephalosporin resistance (3GC-R), carbapenem resistance, and MDR pathogens were assessed for WHO-priority pathogens.

**Results:**

A total of 6575 bacterial isolates were identified, of which 70% (4604) were Gram-negative. The most prevalent Gram-negative organisms were *Klebsiella pneumoniae* (27.0%), *Escherichia coli* (24.3%), and *Pseudomonas aeruginosa* (10.7%). Among Enterobacterales, 3GC-R was detected in 85.1% of *K. pneumoniae* and 81% of *E. coli* isolates. MDR was observed in 34.4% of *K. pneumoniae* and 12.9% of *E. coli*. Carbapenem resistance was reported in 7.4% of *K. pneumoniae* and 1.3% of *E. coli*. Among non-fermenters, *Acinetobacter baumannii* exhibited 18.9% carbapenem resistance and 17.1% MDR, while *P. aeruginosa* showed 38.2% carbapenem resistance and 12.8% MDR. Resistance levels for many key pathogens were notably higher than regional and global averages.

**Conclusions:**

This study reveals concerningly high levels of antimicrobial resistance in Gram-negative bacteria at the tertiary healthcare centre in Guyana. These data add to the regional and global surveillance of antimicrobial resistance. The findings emphasize the need for ongoing surveillance, expanded diagnostic capacity, and implementation of an antimicrobial stewardship programme as part of a multidisciplinary effort to curb antimicrobial resistance in the Caribbean region.

